# Evaluation of Carer Strain and Carer Coping with Medications for People with Dementia after Discharge: Results from the SMS Dementia Study †

**DOI:** 10.3390/healthcare8030248

**Published:** 2020-07-31

**Authors:** Remia Bruce, Wendy Murdoch, Ashley Kable, Kerrin Palazzi, Carolyn Hullick, Dimity Pond, Christopher Oldmeadow, Andrew Searles, Anne Fullerton, Samantha Fraser, Rod Ling, John Attia

**Affiliations:** 1 Hunter New England Local Health District, New Lambton Heights, NSW 2305, Australia; Remia.Bruce@health.nsw.gov.au (R.B.); Wendy.Murdoch@health.nsw.gov.au (W.M.); Carolyn.Hullick@health.nsw.gov.au (C.H.); Anne.Fullerton@health.nsw.gov.au (A.F.); Samantha.Fraser1@health.nsw.gov.au (S.F.); 2 Faculty of Health and Medicine, University of Newcastle, Callaghan, NSW 2308, Australia; Dimity.Pond@newcastle.edu.au (D.P.); Christopher.Oldmeadow@hmri.org.au (C.O.); Andrew.searles@hmri.org.au (A.S.); Rod.Ling@hmri.org.au (R.L.); John.attia@newcastle.edu.au (J.A.); 3 Hunter Medical Research Institute, Newcastle, NSW 2305, Australia; Kerrin.palazzi@hmri.org.au

**Keywords:** carer strain, caregiver strain index, medication management, dementia, dose administration aid, safe medication strategy

## Abstract

This study reports carer strain and coping with medications for people with dementia with an unplanned admission to hospital, and it evaluates the impact of a safe medication intervention on carer coping and carer strain. This was a quasi-experimental pre/post-controlled trial that included a survey of carers about managing medications for people with dementia after discharge. For 88 carers who completed surveys, 33% were concerned about managing medications, and 40% reported difficulties with medication management, including resistive behaviours by people with dementia. Dose administration aids were used by 72% of carers; however, only 15% reported receiving a recent home medicines review by a community pharmacist. High carer strain was reported by 74% of carers. Carer comments described many issues that contributed to high carer stress, as well as their engagement in vigilant activities to maintain medication safety. Strategies that can contribute to carers managing medications and reducing their strain include an increased use of dose administration aids, increased provision of home medicines reviews, and increased education of health professionals to provide adequate support and education about managing medications.

## 1. Introduction

In 2017, it was estimated that there were 196,491 carers of people with dementia in the community in Australia, and most of them were informal carers [1]. The burden of this disease is associated with significant disability and premature mortality [1], and carers/caregivers adopt a substantial role in managing the health needs of their loved ones and coordinating their care [2]. The New South Wales (NSW) Carers (Recognition) Act amended in 2017 defines a carer as a person who provides ongoing and unpaid support to people with disability, terminal illness, chronic illness, mental illness, dementia, or ageing [3], which identifies them as informal carers. People with dementia are frequently prescribed medications and may have associated issues such as polypharmacy, potentially inappropriate medications, complex medication regimens, and multiple medication changes [4,5,6,7], and this places people with dementia at greater risk of medication-related harm [8,9]. Consequently, the carer role can be very demanding, particularly before and after a person with dementia has an unplanned hospital admission. In Australia, one in four people with dementia are reported to be admitted to hospital each year [10].

Recent reviews of published studies of the quality of life and experiences of carers of people with dementia report that the carer’s quality of life is negatively associated with carer burden [11], to the extent that carers of people with dementia have been described as being a hostage of the disease [12]. While carers are adapting to and coping with a hospital admission, they are concerned about the acute illness, exacerbations of behavioural problems, and the potential for deterioration of the person with dementia, and they are often physically and emotionally exhausted. They suffer additional stress immediately prior to the admission, as well as travelling to hospital and disruption to their normal routine. They may also encounter negative interactions with hospital staff, including being ignored, exclusion from decision-making, and not receiving adequate information for and about the person with dementia in hospital [13,14].

A recent review of evidence-based interventions for transitions in care for people with dementia recommended that carers should be prepared and educated about transitions in care (e.g., hospital to home), and included in a family-centred way to help avoid poor outcomes such as readmissions, re-presentations to the emergency department (ED), medication errors, and carer stress [15]. However, hospital discharges at short notice may result in limited opportunities for families to be involved in medication decisions, education, and planning [16].

After patient discharge, carers of people with dementia are frequently responsible for managing complex medication regimens and avoiding medication errors, including identifying possible drug interactions, observing for side effects, sometimes making judgements about (withholding, increasing, decreasing, or discontinuing) medications, monitoring changes in prescriptions, and arranging for continuing supplies of medications [17,18]. Furthermore, they may encounter difficulties in this role including coping with regimen complexity, managing PRN (from the latin ‘pro re nata’ meaning ‘as needed’) medications, behavioural and cooperation issues of people with dementia, changes in medications, and understanding the substitution of generic medications, especially in circumstances where there has been a lack of training and support in medication management [6]. Medication complexity has been described as rapidly changing dose and frequency, medications prescribed for multiple conditions, and complicated administration and storage requirements [16]. People with dementia have been reported to be taking an average of 10 medications in hospital [7]. These factors contribute to their burden and stress [17,18]. Some carers may have access to medication dose administration aids (DAAs; such as dosette boxes or blister packs) to assist them to manage medications for people with dementia, and some may have a home medicines review (HMR) provided by a community pharmacist after the person with dementia is discharged. However, previous studies report that although these forms of carer medication management support are desirable [9], they may not be frequently or routinely provided after discharge [19,20].

The primary aim of this study was to evaluate whether a safe medication strategy (SMS) (intervention) compared with usual care, provided to people with dementia in hospital and at discharge to the community, would reduce readmissions to hospital and re-presentation to ED within three months (results reported separately). This paper reports one aspect of the study: specifically, carer strain and carer coping with medications for people with dementia after discharge home from hospital, and it evaluates the impact of a safe medication intervention for people with dementia on carer coping and carer strain.

## 2. Materials and Methods

### 2.1. Study Design and Setting

This prospective quasi-experimental pre/post-controlled trial was conducted at two regional hospitals (an intervention site and a control site), in the Hunter New England Local Health District (HNELHD) in New South Wales, Australia, over a two-year period (October 2017 to September 2019). The study was conducted in two phases: a pre-intervention phase (Phase 1), and a post-intervention phase (Phase 2). Usual care was delivered at both study sites in phase 1 and at the control site in phase 2. The intervention was delivered at the intervention site in phase 2.

### 2.2. Participant Eligibility

Daily admission reports, provided by the HNELHD, listed all new inpatients over 50 years admitted to the cardiology, general medicine, and general surgical wards. Project staff reviewed these reports to identify potentially eligible participants for the study. Inpatients with an unplanned admission via ED were eligible if they had dementia or cognitive impairment. Admissions from home or residential aged care facilities (RACF) or acute transfers from another ED in the HNELHD were eligible, but inpatient transfers were excluded. Details about screening for and identifying cognitive impairment are published in a separate article [7].

### 2.3. Participant Recruitment

Two study nurses conducted participant recruitment of eligible admissions. It was necessary to seek substitute consent from the person responsible for eligible patients (usually their carer). The NSW Guardianship Act 1987 (Part 5: Substitute Consent: What the law says) [21] hierarchy of persons responsible to determine the appropriate person to approach was used to seek consent for the patient to participate in the study.

Project staff contacted the person responsible, explained that staff had determined the patient had memory and confusion problems, and sought confirmation about whether the patient had these problems at home. If confirmed by the person responsible, they were provided with information about the study and invited to consider providing consent for their relative to participate in the study. The carers were also invited to be a participant in the study, and if they consented, they were advised that they would be contacted three months after the person with dementia was discharged, to participate in a telephone survey about managing medications for people with dementia at home.

### 2.4. Study Sample

A sample size calculation was performed to determine the number of people with dementia required for the primary study outcomes (reduced readmissions to hospital and re-presentation to ED within three months). This study was not powered to detect changes in the secondary (carer) outcomes reported in this article. The study sought to recruit 640 people with dementia; however, only participants who were admitted from and discharged to home (*n* = 523) were likely to have a carer.

### 2.5. The Intervention

**Figure 1:**
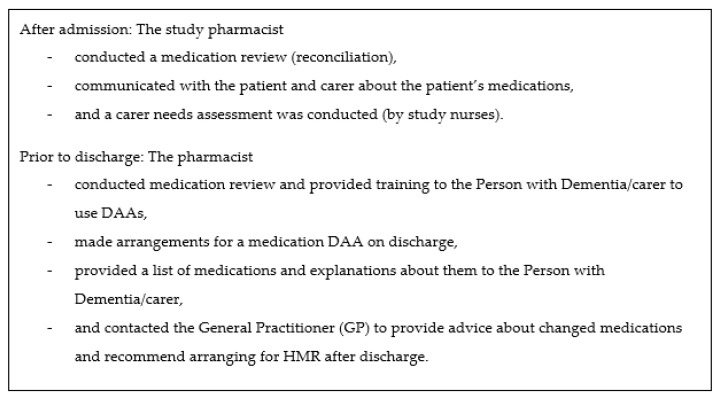
Seven safe medication strategies delivered routinely as a bundle (intervention).

The intervention was a safe medication strategy that was routinely delivered to all people with dementia during phase two of the study at the intervention site. The intervention included seven strategies delivered at admission and at discharge, most of which involved engaging with the carer and the person with dementia (Figure 1). Usual care was provided at both study sites in phase one and at the control site in phase two. Usual care was delivered as individual strategies on an ad hoc basis, rather than as a bundle of strategies delivered routinely. This intervention bundle was designed to be delivered to each participant during phase two at the intervention site. At admission, each participant was visited by the study pharmacist, who conducted a medication reconciliation and communicated with the patient and their carer about the patient’s medications. In addition, a carer needs assessment was conducted. Just prior to discharge, the pharmacist visited each participant again and conducted a medication reconciliation. Then, they provided training to use DAAs, made arrangements for DAAs for the patient after discharge, provided a list of medications and explanations about them to the patient and carer, and contacted the patient’s GP to advise them about medication changes for the patient and to recommend arranging for HMR after discharge.

### 2.6. Instruments

The study instruments were developed by the research team, which included medical officers, specialist aged care nurses, and pharmacists. They were subsequently submitted to an advisory committee with membership that included a carer and person with dementia as well as a broad clinician representation. The instruments were amended in response to the advice received by members of the committee.

#### 2.6.1. Post-Discharge Telephone Questionnaire

The questionnaire was designed to be delivered by telephone after the person with dementia was discharged. It consisted of two parts (Supplementary Files). Part A contained demographic data items and questions about time involved in providing care for their relative with dementia. Part B contained questions about concerns and difficulties coping with managing medications for people with dementia, the use of DAAs, other medications at home after discharge, whether they knew the indications for the medications they were managing for people with dementia, and HMR after discharge, and it also invited comments about the impact of being responsible for managing medications for people with dementia. The Modified Caregiver Strain Index (MCSI) [22] was included in the questionnaire. It is comprised of 13 items designed to measure carer strain after hospital discharge. The MCSI was designed to measure strain associated with carers medication management (originally called medication administration hassles) and is a validated instrument for informal carers [23] with an internal reliability of (α = 0.90) [22]. Permission was provided by the copyright holder to use the Modified Caregiver Strain Index instrument in this study. Some examples of MCSI questions for carers include how frequently they experience disturbed sleep, find caregiving is a physical strain, have changes in their personal plans, experience emotional adjustments or upsetting behaviour, encounter financial strain, and feel completely overwhelmed.

#### 2.6.2. Admission Carer Needs Assessment

The carer needs assessment was a component of the intervention, so it was only conducted for carers at the intervention site during the intervention phase (two) of the study, as soon as practicable after the person with dementia was admitted. It consisted of two parts that were derived from the post-discharge telephone questionnaire to allow for a comparison of data items (coping with medications and carer strain) at admission and after discharge for carers of people with dementia who received the intervention.

Part A was identical to Part A of the telephone questionnaire, and it also contained the MCSI. Part B contained the same items as Part B in the telephone questionnaire except for the items that specifically measured data about the post-discharge period and the MCSI.

### 2.7. Data Collection

The post-discharge telephone carer survey was conducted by the study nurses three months after patient discharge, with carers from both study sites, in phases one and two.

The admission carer needs assessment was conducted by project staff as soon as possible after the person with dementia was admitted and recruited into the study, and it included only carers of people with dementia at the intervention site during phase two of the study.

Study data were collected and managed using REDCap (Research Electronic Data Capture) tools [24] hosted at the Hunter Medical Research Institute (HMRI), Australia.

### 2.8. Data Analysis

Descriptive statistics are presented as count (%) and mean (standard deviation; SD).

The treatment effect at three months post-discharge was analysed for dichotomous (Y/N) outcomes (concern about managing medications, difficulty managing medications, DAA use, HMR reported, still taking prior medications) using logistic regression, and for continuous outcomes (MCSI) using linear regression. Regression modelling variables included phase (one versus two), site (control versus intervention), and an interaction term (phase*site); given adequate response numbers, adjusted modelling included carer characteristics identified as being potentially unbalanced between the sites/phases (carer gender, age, and relationship (child versus other)). For logistic regression, if an adequate model fit was not found due to a low number of responses, crude modelling only was performed; if a separation of model fit was seen due to small numbers, Firth’s penalised likelihood method was applied [25]. In linear mixed modelling, robust standard errors were used to account for minor model fit deviations. Model estimates are presented as the predicted probability or least-squares (LS) mean by phase and site, the odds ratio or mean difference with 95% confidence interval (CI) and *p*-value for phase two versus phase one within each site, the odds ratio or mean difference with 95%CI and *p*-Value for the intervention site versus control site within each phase, and the interaction odds ratio or mean difference with 95%CI and *p*-Value comparing the differences in phase changes between the sites (treatment difference).

Regression modelling includes data from complete cases only; as such, this is not an intention to treat (ITT) analysis, as participants missing outcomes at three months were not included in modelling.

At the intervention site in phase two, the change from admission to three months post-discharge was analysed using logistic mixed modelling for DAA use. Modelling included a fixed effect for timepoint, the covariates described above (given adequate numbers), and a random effect for participants to account for within-subject correlation over time. Model estimates are presented as predicted probability by timepoint and the odds ratio with 95%CI and *p*-Value (treatment difference).

Statistical analyses were programmed using SAS v9.4 SAS Institute Inc, Cary, NC, USA [26]. A priori, *p* < 0.05 (two-tailed) was used to indicate statistical significance. For the MCSI, results were calculated with a possible range of 0–26. Carers who scored seven or more were considered to be suffering a high level of strain.

Carers’ comments about the impact of being responsible for managing medications for people with dementia in the form of text data were categorised using qualitative descriptive analysis. The analytic strategies used were an initial coding/labelling of text data, sorting data to identify topics, identifying categories, identifying commonalities and differences, and deciding generalisations that are true for the data. This was similar to the approach used in a previous qualitative descriptive study [2] and based on the process described by Miles and Huberman and Neergaard et al. [27]. The labelling and coding of data was conducted manually by two researchers (RB, WM) independently, and then reviewed by a senior researcher (AK), and the final analysis was reviewed and consensus was achieved about the findings to be reported.

### 2.9. Ethical Considerations

This study was approved by the Hunter New England Health Human Research Ethics Committee (HREC) (17/06/21/4.08) and University of Newcastle (Australia) HREC (H-2017-0260).

## 3. Results

There were 156 carers who consented to participate in the carer post-discharge telephone survey, which is a consent rate of 29.8% from 523 people with dementia who were admitted from home. Of these, 46 of 80 (58%) carers from the control site and 42 of 76 (55%) carers from the intervention site completed telephone questionnaires. Some carers could not be followed up at three months after discharge (*n* = 68) due to the inability to contact carers, the subsequent admission of people with dementia to RACF, or transfer to a private hospital (19), carers being too stressed to complete the survey, and the death of 25 people with dementia. In addition, 35 carers completed the admission carer needs assessment during the intervention.

### 3.1. Participants

Most carers were female (77%), and the mean age of carers ranged from 61 to 68 years across sites and phases. There were no significant differences between carer’s characteristics at both study sites and in both phases (Table 1). For carers surveyed at the intervention site (phase two) at admission, 66% (21/32) knew about the indications for the medications required for the person with dementia, and this increased to 100% (18/18) at three months after discharge. Overall, (across both sites and phases) at three months after discharge, 94% (81/86) knew about the indications for medications.

### 3.2. Coping with Medications

Overall, 33% (28/86) of carers were concerned about managing medications for people with dementia, and 15% (13/88) were concerned about changes in medications at both sites and in both phases after discharge. Adjusting for carer age, gender, and relationship, there was no significant treatment effect for carer concerns about managing medications at three months after discharge (*p* = 0.630) (Supplementary Tables SA and SB, Additional File S1). Carers were concerned about managing medications, changes in doses, changes in medications, and changes in the appearance of medications. They were uncertain about reasons for medications and which medications should be/should not be given, confused about medication instructions provided, and worried about side effects of medications.

In addition, 40% (34/86) of carers reported that they had difficulties managing medications for people with dementia, and 32.4% (11/34) indicated that they had some difficulty obtaining cooperation from the people with dementia with taking medicines. Adjusting for carer age, gender, and relationship, there was no significant treatment effect for carer-reported difficulties managing medications at three months after discharge (*p* = 0.954) (Supplementary Tables SC and SD, Additional File S1).

### 3.3. Dose Administration Aids (DAAs) Use

In phase two, at the intervention site the use of DAAs by carers was noted to increase from 56% to 72% (Table 2) between admission and after discharge. Adjusting for carer age, gender, and relationship, this difference was not statistically significant: odds ratio 2.82 (95%CI 0.6, 14.1), *p* = 0.193.

**Figure 2:**
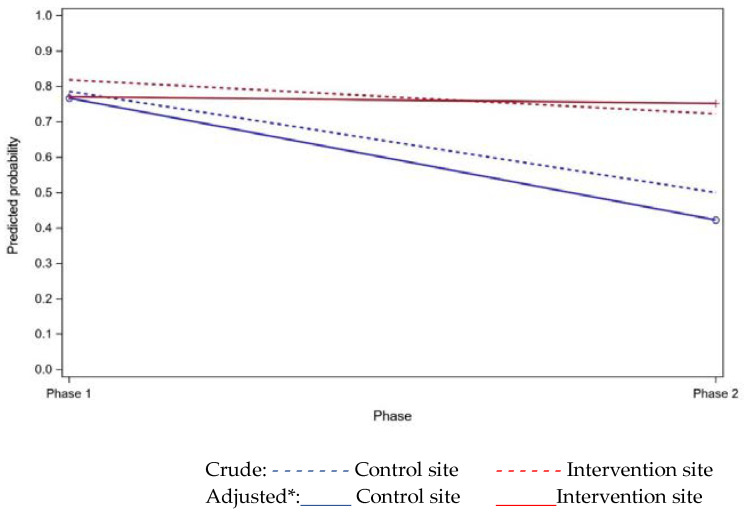
Predicted probability of dose administration aid (DAA) use (three months after discharge) from regression modelling. *Adjusted for carer gender, age, and relationship.

Overall, the use of DAAs after discharge was 72%, and most carers found them to be helpful. Adjusting for carer age, gender, and relationship, there was no significant treatment effect for DAA use at three months after discharge (*p* = 0.203). See Table 2, Figure 2, and Supplementary Table SE, Additional File S1. There was a significant difference between phase one and two at the control site (*p* = 0.028); however, this may represent variation over time found by chance (type 1 error), as this is not the intervention site.

### 3.4. Home Medicines Reviews

Only 15% of carers overall reported that a home medicines review had been conducted by their local pharmacist recently. There was no significant treatment effect for HMR reported by carers at three months after discharge (*p* = 0.185); however, an increased proportion was noted at the intervention site in phase two compared with phase one. See Table 3 and Supplementary Table SF, Additional File S1.

### 3.5. Other Medications

Carers also reported that after discharge, 76% (65/86) of people with dementia were taking other medicines that they already had at home before they went to hospital, and they reported that most of these were medicines for pain or constipation (47.7% and 40% respectively). Adjusting for carer age, gender, and relationship, there was no significant treatment effect for still taking prior medications reported by carers at three months after discharge (*p* = 0.512). See Supplementary Table SG, Additional File S1.

### 3.6. Modified Caregiver Strain Index Results

Results of the MCSI included carers’ reported mean scores ranging from 10 to 12 across the study sites and phases, and overall, 74% (64/86) had high to very high levels of strain (MCSI score of seven or more). Adjusting for carer age, gender, and relationship, there was no significant difference in the MCSI scores between groups due to the intervention (*p* = 0.743); however, a reduced score was noted at the intervention site after discharge in phase two compared to phase one. See Supplementary Tables SH and SI, Additional File S1.

### 3.7. Carer Perceptions of the Impact of Being Responsible for Managing Medicines for People with Dementia

Eighty-six carer participants provided 226 additional comments about the impact of being responsible for managing medicines for people with dementia, and associated concerns and difficulties managing medications at three months after discharge. Comments were labelled and coded into 21 topics that contributed to two categories: the impact on carers and the issues they experienced managing medications for people with dementia (Supplementary Table SJ, Additional File S1).

The positive impact on carers included comments about coping with medications, using DAAs that were helpful, and receiving good medication information and support from primary health professionals (general practitioners and community pharmacists). Less desirable impacts on carers included comments that indicated carers had to cope with potentially serious adverse drug events (ADEs), suffered from high carer stress, and were constantly engaged in providing medication vigilance and monitoring to ensure the safety of people with dementia.

The issues described by carers that contributed to carer stress and carer vigilance included carer burden associated with their own health problems, significant time and financial burden, and carers having difficulty coping with medications. In addition, they described issues with needing information about medications, difficulties with medication packaging, cutting/crushing tablets, medication resistance from people with dementia, confusion and memory problems, not being provided with DAAs, medication changes and complex medication regimes, side effects, medication errors, and problems with getting medication supplies.

Comments about carer vigilance and the monitoring of medications and people with dementia were the most frequent comments provided by carers. There were frequent comments about receiving good medication information and primary healthcare support, using DAAs, difficulty coping with medication changes and complex medications, and carer stress (including needing carer support and not being listened to by health professionals). Although not frequently described, there were some comments about potentially serious adverse drug events from carers. These included potentially toxic doses of potassium, missed doses of medications including Parkinson’s medications that should not be abruptly stopped, potential drug interactions, readmission to hospital due to serious side effects/complications of medications and missed medications, and a duplicate supply of medications.

There were some contrasts between groups in the carers’ comments. Carers in the intervention group did not describe any medication errors in phase two; however, there were 31 descriptions of medication errors from carers in the usual care group. There were almost twice as many comments (19) about receiving good medication information and support from carers in the intervention group compared with the average for usual care (11). Contrasts between groups were also evident for comments by carers in the intervention group at admission compared with the three-month follow-up interview: carers made many more comments in the later interview about the use of, and usefulness of DAAs (12 versus 2) and carer vigilance (15 versus 2).

## 4. Discussion

Carers in this study reported many aspects of managing medications for people with dementia that contributed to carer burden. Carer burden was evident in carers’ descriptions of challenges coping with a substantial time commitment to caring, financial pressure, and their own health problems in addition to their carer responsibilities. The carer role is challenging due to the person with dementia suffering clinically significant confusion, memory loss, and behavioural and psychological symptoms of dementia while concurrently managing pre-existing comorbidities.

In addition to this burden, these carers described activities associated with medication management that constitute carer vigilance, including being responsible for making sure that complex medication regimes are followed, supplies of medications are maintained, changes in medications are adopted, and medication resistance does not result in omitted or mismanaged medications. In addition, carers must monitor people with dementia for side effects, drug interactions, complications, and adverse drug events.

Although carer knowledge about the indications for medications required for people with dementia after discharge at both sites and during both phases was high, at least one-third of carers had concerns and difficulties managing medications after discharge. These issues included not having sufficient information about medications, problems with different packaging and opening medication packages, difficulty cutting/crushing tablets, not using DAAs, complex medication regimes, and monitoring and managing side effects and outcomes of medication errors. Previous studies have reported similar issues for carers involved in managing medications including challenges with maintaining medication supplies (obtaining prescriptions, collecting medications, transport, monitoring supply, access, waiting times, delays, follow up regarding medication details and errors, and medication vigilance), medication administration (packaging issues, reminding people with dementia to take medications, scheduling, cutting tablets, helping with eye drops and creams, uncooperative people with dementia, adapting to medication changes, and the number and frequency of medications), and overall supervision and vigilance of medicine use [17,18,28].

Some carers also described needing to know specific information such as when to withhold medications and how to recognise side effects and adverse effects of medication, and these needs have been reported in previous studies [6,17,28]. Medication changes and complexity, and behavioural problems of people with dementia have also been identified in previous studies as important issues for carers that may contribute to poor therapeutic outcomes for people with dementia, and health professionals may be unaware of this burden [6,17,18].

DAAs were used by 72% of carers after discharge. All of these carers reported they were helpful, which is consistent with other studies that reported DAAs are helpful [9,17]. DAAs are considered to be beneficial because their use can result in fewer missed doses or incorrect doses of oral medication, reduced carer stress, and increased collaboration between primary health clinicians [29,30]. However, some difficulties have been described using DAAs such as not being able to include prescribed oral medications that are ‘only as required’ (PRN) [5]; there are similar problems for medications that require therapeutic dosing or short-term reducing dose medications that cannot be packed in a DAA. Further challenges include that DAAs may not be helpful if the user is forgetful or has impaired dexterity or eyesight; they may be more complicated to use when medication changes occur, they can be costly to adjust and obtain, and they may have up to 10% unintended discrepancies [29].

The proportion of carers who reported HMRs after discharge was only 15%. One of the reasons for conducting HMRs after discharge is to reconcile new medications and recommended medication changes with medications used prior to admission to hospital. Of the carers in this study, 76% reported the continued use of pre-admission medicines (mostly medications for pain and constipation), and this raises issues about possible drug interactions, duplication, and polypharmacy. HMR can reduce polypharmacy through the identification of duplicate medications and potential drug interactions, improvement of regimes and pharmacokinetics (movement of a drug through the body including absorption, distribution, metabolism, and excretion) or irregular dosages, and facilitation of the appropriate de-prescribing of medications with adverse effects or high anticholinergic burden. Additional benefits may include reduced confusion associated with different packaging (including generic medications), addressing carers questions about continuing to use medications prescribed prior to admission to hospital and the potential side effects of medications, and determine missed/missing doses of medications in DAAs. It can also identify over the counter medications that do not require a prescription, and consequently, primary health professionals may not be aware they are being used. There is scope to increase the provision of HMRs for people with dementia after discharge, which has been reported to be below 10% among adults >45 years in Australia [20]; this is recommended by several previous studies [9,17,20,31] and recent reports [8,32].

There were 52 comments about receiving good medication information and support from primary health professionals after discharge. While this support is helpful to ensure adequate information, supply, and dispensing of medications; it is not a substitute for an HMR, which is likely to ensure better medication safety by identifying potential issues.

There were 53 comments that described high carer stress in this study associated with the carer role, which included coping with and managing medication changes that necessitate being vigilant about medications for the person with dementia. Carer stress has been identified in other studies of carers of people with dementia, and it is particularly associated with medication changes and complex regimens and uncooperative people with dementia [5,6,18]. In addition, some carers described clinically significant potentially serious adverse drug events (ADEs). Some carers advised that they were too stressed to complete the survey, and many had high MCSI scores after discharge. Not being heard and feeling excluded from decision making also contributed to carer stress, and this issue has been reported in previous studies in acute care settings [13,14,17]. Early and continued carer consultation, in conjunction with strategies for providing increased carer respite and support to manage medications for people with dementia, would contribute to alleviating carer stress [30].

One of the key findings of this study was that carers frequently described engaging in carer vigilance and monitoring of medications for people with dementia. Carer vigilance has been identified in only a few previous studies where carers have been described as providing constant surveillance of the person with dementia in order to keep them safe [12], checking prescriptions for accuracy and querying changes in medications [5,33], seeking information about medicines and their side effects, and ensuring people with dementia took medicines on time [18].

The combination of carer burden, carer vigilance and monitoring, difficulty coping with medication changes, complex medications and regimes, and potentially serious drug events all contributed to carer stress and highlight their need to be listened to and adequately supported. Previous studies have also described carers as having a sense of guilt or failure if they could not manage medications and recognise changes in the health status of people with dementia that would suggest side effects of medications [34]. Other studies have also reported that carers do not receive adequate support [11,17,34].

There was no significant effect of the intervention on carers in this study for any of the variables measured in the survey, and this may be due to the small numbers of carer participants; however, the differences reported for use of DAAs, the provision of HMR, and reduced carer strain suggest clinically important differences between phase one and two at the intervention site. A review of previous intervention studies designed to measure transitions in care for people with dementia reported that effective interventions involve the carer in the planning, education, and communication of information to help avoid poor outcomes such as unplanned readmissions, re-presentation to ED, medication errors, and carer stress [15].

### 4.1. Limitations

The authors acknowledge that this analysis was done on a small study sample of carers of people with dementia, and this reduced the ability to detect the effect of the intervention on carers in this study. The study sample was further affected by attrition at three months after discharge, and consequently, the results may not be generalisable to other carers of people with dementia after discharge. In addition, there was potential for bias due to self-reported data, recall bias, and responder bias for attrition, and so the results are the perceptions of this sample of carers. It is important to recognise that the recruitment of participants in this study was very complex due to the lack of documentation of cognitive impairment, which necessitated screening patients for cognitive impairment and required the carer to confirm that the patient had confusion or memory problems at home. So, the cognitive status of unplanned admissions may often be undetected. This may be due to a general reluctance in the community and among health professionals to use terms such as ‘dementia’, ‘cognitive impairment’, or ‘Alzheimer’s’ because of the perceived stigma associated with these terms [35,36]. In addition, there is no routine process to alert health professionals about cognitive impairment within the existing hospital electronic monitoring and communication systems, and consequently, even if it is identified during an acute admission, the information may not be considered by health professionals when they engage with carers.

### 4.2. Recommendations for Clinical Practice

The authors recommend the following clinical practices to address the issues identified in the results of this survey.

1. Routine assessment of carer strain should be conducted for admissions with cognitive impairment and support strategies offered.
2. Health professionals need training* about recognising and responding to carer strain and the unmet need for carer support in their role managing medications for people with dementia and the associated impact, including carer vigilance and monitoring, and carer stress.
3. Medication dose administration aids are recommended to assist carers to manage medications for people with dementia effectively.
4. Home medicines reviews after discharge are recommended for people with dementia who have an unplanned admission to hospital to support carers in their role as medication managers.

*There are free training courses available for health professionals including Dementia Training Australia (https://www.dta.com.au/) and a massive open online course from the Wicking Dementia Centre (https://www.utas.edu.au/wicking/understanding-dementia).

These recommendations are consistent with the recent recommendations from Dementia Australia [8,37], and they are based on a report from the Australian Institute of Health and Welfare [32] and other Australian research [9].

## 5. Conclusions

Carers in this study reported having a good understanding of the indications for medications for people with dementia, using DAAs to manage medications and receiving good support from primary health care clinicians. Despite this, carers reported clinically significant issues associated with managing medications for people with dementia, particularly medication changes and complex medication regimes, and having inadequate information and support for managing medications, including not being provided with HMRs. The impact of these issues resulted in high carer stress, a high level of carer vigilance and monitoring, and occasions of adverse drug outcomes and readmission for people with dementia. Informal carers have a critical and challenging role managing medications for people with dementia, and they require adequate support and responses from health professionals to assist them in this role and to maintain medication safety and minimise the risk of harm to people with dementia.

## Figures and Tables

**Table 1:** Carer participant characteristics.

Characteristic	Class/Statistic	Phase 1 Control (*n* = 49)	Phase 1 Intervention (*n* = 40)	Phase 2 Control (*n* = 31)	Phase 2 Intervention (*n* = 36)
Gender	Male	5 (18%)	5 (23%)	2 (11%)	12 (34%)
	Female	23 (82%)	17 (77%)	16 (89%)	23 (66%)
	Missing	21	18	13	1
Age	Mean (SD)	64 (9)	61 (9)	63 (11)	68 (13)
	Missing	21	19	14	1
Relationship	Spouse/Partner/Sister/Cousin	12 (43%)	6 (27%)	7 (39%)	18 (51%)
(combined)	Child/Daughter in law	16 (57%)	16 (73%)	11 (61%)	17 (49%)
	Missing	21	18	13	1

**Table 2:** Dose administration aids use.

Characteristic	Response	Phase 1 Control (*n* = 49)	Phase 1 Intervention (*n* = 40)	Phase 2 Control (*n* = 31)	Phase 2 Intervention (*n* = 36)
**Admission**	-	-	-	-	-
Are you using medicine dose administration aids such as a blister pak or dosette?	No	-	-	-	14 (44%)
	Yes	-	-	-	18 (56%)
	Missing	49	40	31	4
**After Discharge**	-	-	-	-	-
Are you using medicine dose administration aids such as a blister pack or dosette?	No	6 (21%)	4 (18%)	9 (50%)	5 (28%)
	Yes	22 (79%)	18 (82%)	9 (50%)	13 (72%)
	Missing	21	18	13	18
Do you find this helpful?	No	1 (4.5%)	-	-	-
	Yes	21 (95%)	18 (100.0%)	9 (100.0%)	13 (100.0%)
	Missing	27	22	22	23

**Table 3:** Home medicines reviews.

Characteristic	Response	Phase 1 Control (*n* = 49)	Phase 1 Intervention (*n* = 40)	Phase 2 Control (*n* = 31)	Phase 2 Intervention (*n* = 36)
Have you had a home medicines review done by your local pharmacist at home recently?	No	22 (81%)	20 (91%)	16 (89%)	13 (76%)
	Yes	5 (19%)	2 (9.1%)	2 (11%)	4 (24%)
	Missing	22	18	13	19

## Supplementary Materials

The following are available online at https://www.mdpi.com/2227-9032/8/3/248/s1, Supplementary Tables SMS Dementia Study Carers, Additional File S1.
